# Biodiversity estimates and ecological interpretations of meiofaunal communities are biased by the taxonomic approach

**DOI:** 10.1038/s42003-018-0119-2

**Published:** 2018-08-14

**Authors:** Francesca Leasi, Joseph L. Sevigny, Eric M. Laflamme, Tom Artois, Marco Curini-Galletti, Alberto de Jesus Navarrete, Maikon Di Domenico, Freya Goetz, Jeffrey A. Hall, Rick Hochberg, Katharina M. Jörger, Ulf Jondelius, M. Antonio Todaro, Herman H. Wirshing, Jon L. Norenburg, W. Kelley Thomas

**Affiliations:** 1 Department of Biology, Geology and Environmental Science, University of Tennessee at Chattanooga, 615 McCallie Avenue, Chattanooga, TN 37403 USA; 2 Hubbard Center for Genome Studies, Department of Molecular, Cellular, and Biomedical Sciences, University of New Hampshire, 35 Colovos Road, Durham, NH 03824 USA; 3 Department of Mathematics, Plymouth State University, MSC29, 17 High Street, Plymouth, NH 03264 USA; 4 Centre for Environmental Sciences, Hasselt University, Campus Diepenbeek, Agoralaan Gebouw D, 3590 Diepenbeek, Belgium; 5 Dipartimento di Medicina Veterinaria, University of Sassari, via Muroni 25, 07100 Sassari, Italy; 6 Departmento de Sistemática y Ecología Acuática, El Colegio de la Frontera Sur, Unidad Chetumal, Av. Centenario Km. 5.5 Chetumal Quintana Roo, 77014 Chetumal, Mexico; 7 Centro de Estudos do Mar, Universidade Federal do Paraná, Av. Beira-Mar, s/n, Pontal do Sul, PO Box 61, 83255-976 Pontal do Paraná, PR Brazil; 8 Department of Invertebrate Zoology, Smithsonian National Museum of Natural History, 10th St. & Constitution Ave NW, Washington, DC 20560 USA; 9 Department of Biological Science, University of Massachusetts Lowell, Olsen Hall 414, 198 Riverside St., Lowell, MA 01854 USA; 10 Department of Biology, Ludwig-Maximilians–University of Munich, Großhaderner Str. 2, 82152 Planegg-Martinsried, Munich, Germany; 11 Swedish Museum of Natural History, POB 5007, SE-104 05 Stockholm, Sweden; 12 Department of Life Sciences, University of Modena & Reggio Emilia, Via G. Campi 213/d, 41125 Modena, Italy

## Abstract

Accurate assessments of biodiversity are crucial to advising ecosystem-monitoring programs and understanding ecosystem function. Nevertheless, a standard operating procedure to assess biodiversity accurately and consistently has not been established. This is especially true for meiofauna, a diverse community (>20 phyla) of small benthic invertebrates that have fundamental ecological roles. Recent studies show that metabarcoding is a cost-effective and time-effective method to estimate meiofauna biodiversity, in contrast to morphological-based taxonomy. Here, we compare biodiversity assessments of a diverse meiofaunal community derived by applying multiple taxonomic methods based on comparative morphology, molecular phylogenetic analysis, DNA barcoding of individual specimens, and metabarcoding of environmental DNA. We show that biodiversity estimates are strongly biased across taxonomic methods and phyla. Such biases affect understanding of community structures and ecological interpretations. This study supports the urgency of improving aspects of environmental high-throughput sequencing and the value of taxonomists in correctly understanding biodiversity estimates.

## Abstract (web summary)

Francesca Leasi et al. report a comparison of approaches for estimating meiofauna diversity, including metabarcoding and other morphological-based taxonomy methods. They show bias in estimates based on methods and phyla, highlighting the need for a standard procedure for assessing biodiversity.

## Introduction

We are currently experiencing the fastest loss of biodiversity since the extinction of dinosaurs 66 million years ago^1^. Meanwhile, the role of biodiversity in maintaining ecosystems should be considered prominent in climate change science and policy^2^. Awareness of current extinction rates has led to the establishment of large-scale conservation programs whose effectiveness relies upon the ability to thoroughly and rapidly assess biodiversity and ecosystem health^3^. Thus, there is a need for methods that accurately and efficiently appraise ecosystem biodiversity and temporal variations following natural or anthropogenic events. The improvement of molecular techniques in recent years has allowed for the development of genetic methods that help increase the rate and accuracy of species identification^4^. Large-scale DNA barcoding (metabarcoding) has become especially useful for analyses of environmental collections (sediment, water, soil, gut content, etc.) for which biodiversity assessment is implemented at the community level^5^. Metabarcoding enables assessment of biodiversity where the molecular taxonomy can be communicated unambiguously. However, those sequences alone are not linked to a specific morphology. By contrast, morphology-based taxonomy is inherently linked to the characteristics of the organisms but it is extremely labor-intensive and difficult to communicate unambiguously.

Metabarcoding is particularly useful to identify small-sized species, such as the meiofauna, for which morphological identification is time-consuming and requires rare taxonomic expertise^6^. Moreover, organisms typically excluded from morphological analyses, such as pieces of animals, juvenile stages, rare taxa, and cryptic species may be detected by metabarcoding^6,7^. Meiofauna include a heterogeneous collection of small (<1 mm) marine benthic invertebrates of almost all animal phyla, with a wide range of functional groups that have key roles in benthic energy flow^8,9^. Metabarcoding has been applied to meiofauna in a number of cases usually involving intentionally preserved samples for molecular analyses^10,11^. In contrast, morphology-based taxonomy for many groups of meiofauna requires observations on fresh/live specimens. Consequently, metabarcoding allows the reconstruction of community compositions from deep sea, polar regions, cold seeps, hydrothermal vents, etc., where investigation of live organisms would be difficult or impossible^7,11^. However, only a few studies have tested the relationship between metabarcoding and morphological assessment of diversity. For practical reasons, such studies have focused on mock communities of animals with an exoskeleton such as nematodes and copepods^12,13^. The other >20 phyla inhabiting the marine interstitial environment, most of which are soft-bodied, have been ignored.

The present study aims to investigate metabarcoding and morphological-based assessment of diversity across members of seven, mostly soft-bodied animals, phyla. In this assessment, we compared the results of a metabarcoding analysis across numerous samples with morphological and molecular assessments of individual specimens from those samples. The goal was to test for biases inherent in the taxonomic approach applied, and whether such biases affect broad ecological interpretations at the community level. Specifically, we tested whether different approaches result in significant differences in estimates of biological diversity; if such differences are consistently present in all seven phyla; and if the approach affects the ecological conclusions of a field study. Results showed that the taxonomic approach significantly biases biodiversity estimates, and such biases are inconsistent across phyla. Therefore, community structures and ecological correlations within and among community shift according to the taxonomic method applied.

## Results

### Environmental conditions

**Fig. 1:**
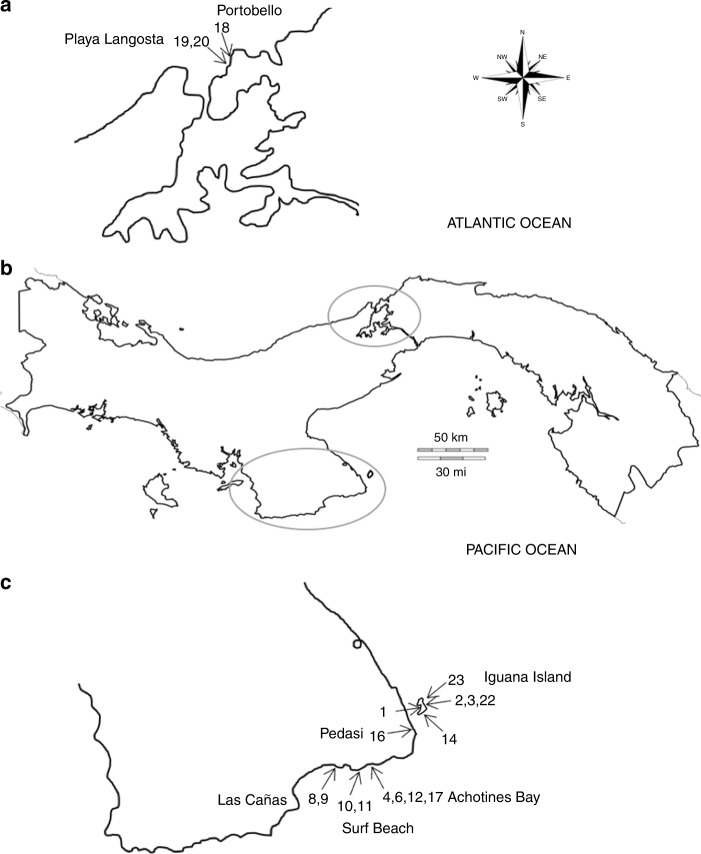
Map indicating the investigated area (Panama). Sample sites are indicated by arrows and numbers. **a** Detail showing Atlantic sampling sites; **b** general view of Panama with investigated areas circled; **c** detail of Pacific sites

Samples were collected from 19 sites located in Panama (Fig. 1). The salinity^14^ value was 38 for most of the samples except for one site with a salinity at 31 (station 9) and two sites at 36 (stations 10, 11). Sedimentological analyses indicated heterogeneous sampling, with a grain size ranging from fine to very coarse and distribution of size from well sorted to very poorly sorted. The sediments from three samples (stations 2, 14, 23) were too coarse (mean grain size >2 mm) to analyze additional sedimentological parameters using our procedure (see additional tables for a list of sampled sites and associated environmental parameters^15^).

### Species richness

**Fig. 2:**
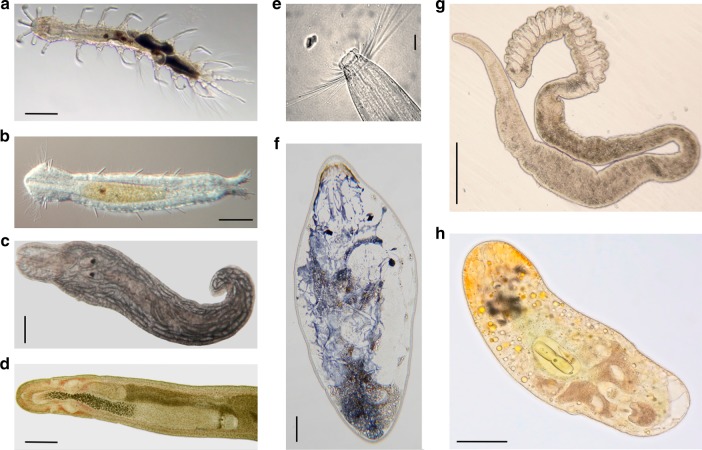
Photos of some representative meiofaunal organisms collected from Panama. **a** Annelida, *Nerilla* sp. (scale bar: 100 µm). **b** Gastrotricha, *Dactylopodola* sp. (scale bar: 50 µm). **c** Mollusca, *Rhodope* sp. (scale bar: 100 µm). **d** Nemertea, *Ototyphlonemertes* cf. *erneba* anterior end (scale bar: 200 µm). **e** Nematoda, *Steineria* sp. 2 close up of head (scale bar: 10 µm). **f** Platyhelminthes Rhabdocoela, *Polycystis* sp. (scale bar: 100 µm). **g** Platyhelminthes Proseriata, *Polystyliphora* sp. (scale bar: 100 µm). **h** Xenacoelomorpha, *Isodiametra* sp. (scale bar: 100 µm)

A total of 835 individuals belonging to seven phyla and allocated to 187 morphotypes were collected from the 19 sampled sites (Fig. 2). Specifically, 20 morphotypes belonged to the phylum Annelida, 37 were Gastrotricha, 22 Mollusca, 37 Nemertea, 23 Platyhelminthes, 38 Nematoda, and 10 morphotypes belonged to the phylum Xenacoelomorpha (Table 1; see additional tables for a detailed list of morphotypes^15^).

**Table 1:** Table summarizing the sample size (number of specimens, 18S rRNA sequences, and genetic reads) and richness (number of morphotypes, entities, OTUs, eOTUs, and sequence variants) obtained from each taxonomic method

	Single individuals					Metabarcoding			
Phylum	Specimen	Morphotype	18S rRNA Sequence	Entity	OTU	Reads eOTU	eOTU	Reads SV	SV
Annelida	163	20	133	23	27	9248	178	9893	63
Gastrotricha^a^	133	37	107	27	38	265	20	1083	12
Mollusca^a^	96	22	81	6	17	4751	36	4423	22
Nematoda	108	37	73	2	37	7818	155	8243	89
Nemertea^a^	94	23	83	9	34	235	12	242	8
Platyhelminthes	208	38	163	42	48	5666	120	5993	87
Xenacoelomorpha^a^	33	10	28	7	10	4223	26	640	12
Total	835	187	668	116	211	32,206	547	29,279	293

Phyla for which richness recognized with morphology-based taxonomy is higher than richness recognized with at least one metabarcoding approach are indicated by superscript “a”

Morphotypes were identified with comparative morphology; entities were recovered via DNA taxonomy (GMYC model) from single specimens

*OTU* operational taxonomic units recovered via 18S rRNA sequences from single specimens, *eOTU* operational taxonomic units recovered via metabarcoding, *SV* sequence variants recovered via metabarcoding

DNA taxonomy obtained with the Generalized Mixed Yule Coalescent (GMYC) model revealed 116 evolutionary independent entities, among which 23 Annelida, 27 Gastrotricha, 6 Mollusca, 2 Nematoda, 9 Nemertea, 42 Platyhelminthes, and 7 Xenacoelomorpha. Results obtained by using the GMYC species delineation model were not significant (Annelida, Mollusca, Nematoda, *p* = 0.773; Nemertea, *p* = 0.205; Xenacoelomorpha, *p* = 0.199), except for species of Gastrotricha (*p* = 0.0015) and Platyhelminthes (*p* = 0.016) whose 18S datasets seemed to better fit the GYMC model (see additional tables for a detailed list of evolutionary independent entities^15^).

The same genetic dataset as described above was considered to determine the number of Operational Taxonomic Units (OTUs) for each phylum. A total of 211 OTUs were obtained, among which were 27 Annelida, 38 Gastrotricha, 17 Mollusca, 37 Nematoda, 34 Nemertea, 48 Platyhelminthes, and 10 Xenacoelomorpha (see additional tables for a detailed list of OTUs assessed on single specimens^15^).

Meanwhile, biodiversity was assessed with metabarcoding for each of the 19 investigated sites. Taxonomic units obtained with the metabarcoding approach were categorized by groups in order to remove all organisms that are not meiofaunal invertebrates (e.g., fungi, vertebrates, plants, algae, known big invertebrates, and protists). The final dataset consisted of 120133 genetic reads and 1345 environmental OTUs (eOTUs) distributed in 20 phyla. The richest phylum was Arthropoda with 699 eOTUs, most of which (659) were Copepoda. By pruning the dataset to the seven focal phyla, we obtained a total of 32206 genetic reads and 547 eOTUs. In order of richness, 178 eOTUs were Annelida, 155 Nematoda, 120 Platyhelminthes, 36 Mollusca, 26 Xenacoelomorpha, 20 Gastrotricha, and 12 Nemertea (see additional tables for a detailed list of eOTUs assessed with metabarcoding^15^).

The same initial genetic dataset was analyzed and filtered to obtained sequence variants. Compared to eOTUs, we obtained a similar number of genetic reads (127,002 for the total meiofaunal community dataset) but these clustered in a lower number of unique units (496 sequence variants). The seven focused phyla were present with a total number of 29279 genetic reads and 293 sequence variants. In contrast to results obtained with eOTU, the richest taxon was Nematoda (89 sequence variants), followed by Platyhelminthes (87 sequence variants), Annelida (63 sequence variants), Mollusca (22 sequence variants), Gastrotricha and Xenacoelomorpha (both present with 12 sequence variants), and Nemertea with 8 sequence variants (see additional tables for a detailed list of sequence variants^15^).

**Fig. 3:**
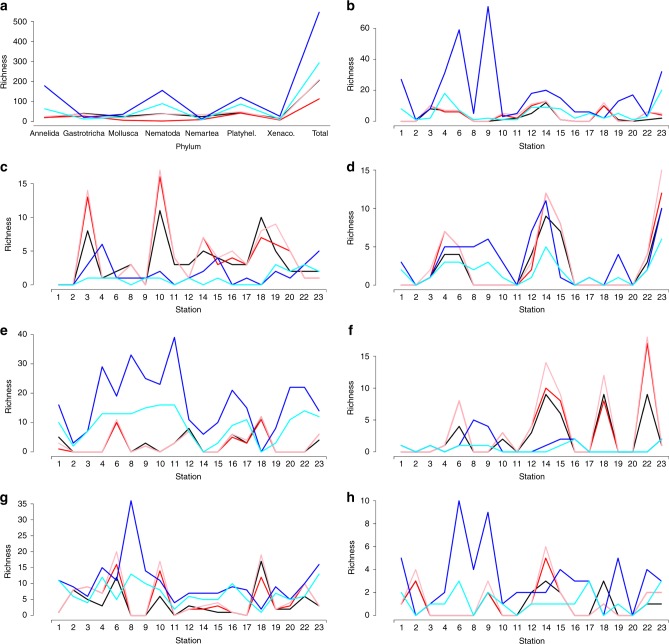
Plots showing the richness assessed via each taxonomic method. **a** Total richness measured in all sampled sites from each phylum. **b**–**h** Richness for each taxon at each investigated site, including Annelida (**b**), Gastrotricha (**c**), Mollusca (**d**), Nematoda (**e**), Nemertea (**f**), Platyhelminthes (**g**), and Xenacoelomorpha (**h**). Morphotypes (black line) are identified with comparative morphology; evolutionary independent entities (red line) are recovered via DNA taxonomy (GMYC model) from 18S rRNA sequences (V1–V2 regions) of single specimens; OTUs (pink line) recovered via 18S rRNA sequences (V1–V2 regions) of single specimens; eOTUs (dark blue line), recovered via metabarcoding 18S rRNA (V9 region); sequence variants (light blue line), recovered via metabarcoding 18S rRNA (V9 region)

Statistical analyses showed that the taxonomic method used to assess biodiversity was a significant predictor of whole community richness (Wald *χ*^2^ = 141.61, *p* < 0.0001). Based on p-values and estimated confidence intervals associated with the pairwise comparisons of richness assessed by different methods, we identified that morphotypes, OTUs, and evolutionary independent entities obtained with GMYC model on single individuals were not statistically different to one another (morphotype versus OTU, *p* = 0.353; morphotype versus entity, *p* = 1; OTU versus entity, *p* = 0.375). Next, we identified that eOTUs and sequence variants obtained from environmental samples were statistically different from one another (*Z* = 3.319, *p* = 0.008). Lastly, species richness estimated with both eOTUs and sequence variants were independently different from morphological units (*p* < 0.0001 and *p* = 0.029, respectively), OTUs (*p* < 0.0001 and *p* = 0.0297, respectively), and evolutionary independent entities (*p* < 0.0001). Richness assessed by each taxonomic method is shown in Table 1 and Fig. 3.

Pairwise tests for richness by phylum independently revealed significant differences only for Annelida and Nematoda (all *p* < 0.05 as specified below), although, because of the large number of comparisons, false negatives are quite possible. Richness of Annelida significantly differed between eOTUs and the three methods based on single individuals, namely morphotypes (*Z* = 5.258, *p* = 0.0001), OTUs (Z = −5.198, *p* = 0.0001), and evolutionary entities (*Z* = −7.803, *p* < 0.0001). For Nematoda, significant differences in predicted richness were observed between evolutionary entities and both eOTUs (*Z* = −6.191, *p* < 0.0001) and sequence variants (*Z* = −4951, *p* = 0.0004).

The total number of species belonging to the seven phyla estimated in the sampled area spanned between 365 and 609 depending on the species estimator function applied (chao2 and jack1) to the two taxonomic datasets considered (morphological units and sequence variants; (see ref. ^15^ for values of richness estimation obtained using Chao2 and Jackknife algorithms).

### Ecological conclusions across different richness estimates

The correlations between richness and environmental parameters differed across taxonomic methods. Specifically, for the datasets based on single individuals, our final model inferred that sample size (number of individuals or genetic sequences; Wald *χ*^2^ = 51.01, *p* < 0.0001), depth (Wald *χ*^2^ = 6.38, *p* = 0.011), grain size sorting (Wald *χ*^2^ = 4.91, *p* = 0.027), and the dichotomous (dummy) variable distinguishing morphology and one of the two DNA taxonomy methods (OTU) from the other (evolutionary independent entity; Wald *χ*^2^ = 9.71, *p* = 0.002) were statistically significant predictors of richness. Interestingly, none of the interactions with the dichotomous variable were found to be statistically significant (interaction between depth and dummy: Wald *χ*^2^ = 1.32, *p* = 0.25; between sorting and dummy: Wald *χ*^2^ = 0.024, *p* = 0.88; between sample size and dummy: Wald *χ*^2^ = 0.75, *p* = 0.39). For metabarcoding analyses, on both selected phyla and the whole meiofaunal communities, our final model inferred that only the sample size (number of genetic reads; Wald *χ*^2^ = 24.55, *p* < 0.0001; Wald *χ*^2^ = 7.74, *p* = 0.0054, respectively), the dichotomous (dummy) variable distinguishing the two metabarcoding approaches (Wald *χ*^2^ = 27.34; *p* < 0.0001; Wald *χ*^2^ = 131.25; *p* < 0.0001, respectively), and the interaction between the dummy and the sample size (Wald *χ*^2^ = 5.63; *p* = 0.0177; Wald *χ*^2^ = 17.53; *p* < 0.0001, respectively) were statistically significant predictors of richness.

### Community structure

Values of Jaccard dissimilarity were comparable across the five taxonomic methods when biological communities were sampled from similar habitats (either littoral, sublittoral, or offshore). However, pairwise comparisons among the three investigated habitats showed values of Jaccard that were inconsistent across taxonomic methods (see additional tables for values of Jaccard dissimilarity^15^).

#### Ecological conclusions across different community structure estimates

**Fig. 4:**
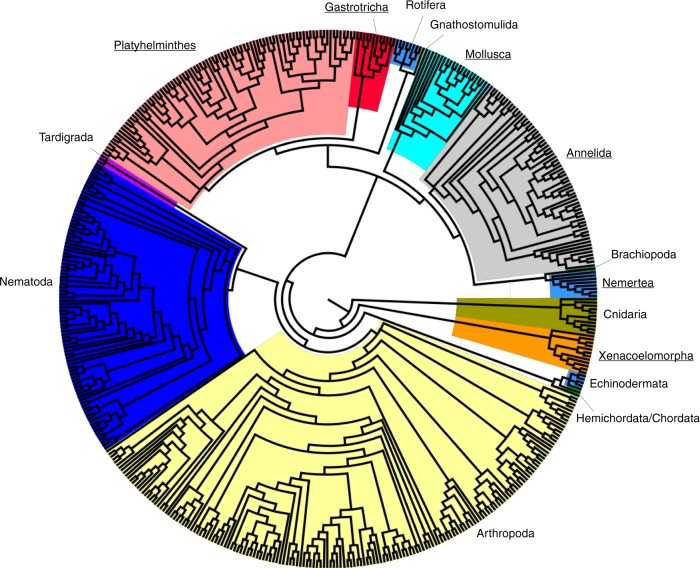
Maximum likelihood constrained phylogeny based on sequence variants showing the taxonomic composition of the whole meiofaunal dataset in the sampled area. The focal phyla are underlined

The results of PERMANOVA suggested that the applied methodology strongly biased the correlations between community compositions and environmental parameters (see additional tables for results obtained with PERMANOVA^15^). Specifically, for morphological taxonomy there were significant correlations between the community structure and depth (*p* = 0.003), mean grain size (*p* = 0.039), and the sedimentological parameter kurtosis (*p* = 0.027); and a nearly significant correlation with (iv) salinity (*p* = 0.082). Community compositions obtained via OTU showed a nearly significant correlation with depth (*p* = 0.064). For the metabarcoding datasets on focal phyla, a nearly significant correlation was found between both eOTU and sequence variant communities and the mean grain size (p-values = 0.099 and 0.087, respectively). When the whole meiofaunal community was analyzed with metabarcoding (15 phyla; Fig. 4), significant correlations were present between eOTUs and both mean grain size (*p* = 0.039) and the sedimentological parameter skewness (*p* = 0.039), and nearly significant correlations between eOTU communities and depth (*p* = 0.098). Sequence variant whole community structures were significantly correlated to depth (*p* = 0.05), and nearly significant correlated to the mean grain size (*p* = 0.069).

### Phylogenetic diversity

**Fig. 5:**
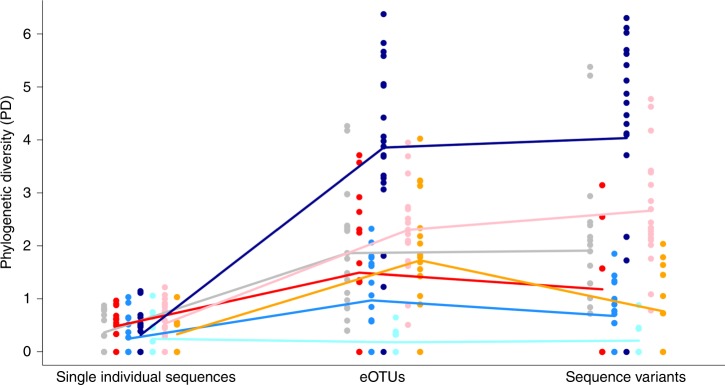
Differences in the values of phylogenetic diversity for each phylum across three taxonomic methods (single individual 18S rRNA sequences, eOTUs, sequence variants). Phylogenetic diversity quantifies the minimum total length of all the phylogenetic branches required to span a given set of taxa on the phylogenetic tree. Lower values of phylogenetic diversity correspond to shorter phylogenetic branches. Lines show average values for each investigate phylum; dots show distinct values of phylogenetic diversity. Colors are assigned as follow: Annelida in gray; Gastrotricha in red; Mollusca in medium blue; Nematoda in dark blue line, Nemertea in light blue; Platyhelminthes in pink; Xenacoelomorpha in orange

Our fitted model inferred that phylum (*F* = 43.15, df = 6, *p* < 0.0001), taxonomic method (*F* = 103.63, df = 2, *p* < 0.0001), and the interaction between phylum and method (*F* = 9.48, df = 12, *p* < 0.0001) were all statistically significant predictors of phylogenetic diversity (Fig. 5; see additional tables for values of phylogenetic diversity measured for each phylum and sampled site^15^). For each phylum, we identified significant differences only between the phylogenetic diversity obtained from single individual datasets and the one obtained from one or both metabarcoding datasets (all associated p-values < 0.05 as specified below), and no significant differences between the two metabarcoding approaches (all associated *p*-values = 1; Fig. 5). In detail, the values of phylogenetic diversity for species of Annelida, Nematoda, Platyhelminthes, and Xenacoelomorpha significantly differed between single individual datasets and both metabarcoding approaches (all associated p-values < 0.0001). The phylogenetic diversity of Mollusca significantly differed between single individual dataset and eOTU dataset (*p* = 0.0214). The phylogenetic diversity of Gastrotricha and Nemertea did not differ significantly across methods (all associated *p*-values = 1).

Similar results were obtained for both Mean Phylogenetic Diversity (MPD) and Mean Nearest Taxon Distance (MNTD). The ANOVA model fit to MPD data exhibited a lack of Normality in the residuals, therefore, a robust ANOVA model was pursued. The fitted results and their associated hypothesis tests based on this robust model were similar to those obtained via an ordinary least-squares regression, and we can be confident that ANOVA results based on the ordinary least-squares model are valid, that phylum, method, and the interaction between phylum and method were all significant predictors of MPD (*p* < 0.0001 for all). Specifically, our fitted model identified that phylum (*F* = 15.36, df = 6, *p* < 0.0001), method (*F* = 64.51, df = 2, *p* < 0.0001) and the interaction between phylum and method (*F* = 4.21, df = 12, *p* < 0.0001) were both statistically significant predictors of MPD. Similarly, our fitted model identified that phylum (*F* = 15.36, df = 6, *p* < 0.0001), method (*F* = 64.51, df = 2, *p* < 0.0001), and the interaction between phylum and method (*F* = 4.21, df = 12, *p* < 0.0001) were all statistically significant predictors of MNTD.

#### Ecological conclusions across different phylogenetic diversity estimates

Among all the investigated ecological parameters and across all taxonomic methods, only one sedimentological parameter (sorting) was found to be a significant predictor of phylogenetic diversity (*p* = 0.0488). However, the relationship between phylogenetic diversity and sorting depended on the animal phylum in question; the interaction between sorting and phylum was a significant predictor of phylogenetic diversity (*F* = 3.0765, df = 6, *p* = 0.0062).

### V1–V2 and V9 primer region comparison

In all the investigated phyla, the average percent identity of the V1–V2 variable region of the 18S rRNA was higher compared to the V9 variable region^16^. Specifically, comparison of 872 18S sequences of Annelida show 0.63 identity for the V9 region and 0.70 identity for the V1–V2 region. Other phyla include Gastrotricha (112 sequences; 0.54 identity for V9, 0.80 for V1–V2), Mollusca (751 sequences; 0.59 identity for V9, 0.70 for V1–V2), Nematoda (1107 sequences, 0.46 identity for V9, 0.65 for V1–V2), Nemertea (133 sequences, 0.72 identity for V9, 0.79 for V1–V2), Platyhelminthes (1154 sequences, 0.67 identity for V9, 0.70 for V1–V2), and Xenacoelomorpha (76 sequences, 0.48 identity for V9, 0.57 for V1–V2).

## Discussion

Here, we have shown estimates of meiofauna biodiversity obtained via different methods, namely morphology-based taxonomy, DNA taxonomy, and metabarcoding. Statistical analyses revealed that the taxonomic method used to assess biodiversity is a strong predictor of biodiversity, which was measured in species richness, community structure, and phylogenetic diversity. In addition to revealing that biodiversity estimates can be biased by the methodology used to define taxonomic units, we have shown that different phyla respond differently to such a bias. Therefore, predictions of species composition within communities change, greatly affecting ecological conclusions.

Previous studies using metabarcoding generally report higher species richness compared to morphological taxonomy^17^. This is likely due to the observation of genotypes of hidden taxa, such as cryptic species, juvenile forms not linked to a morphology, and sequencing artifacts^18^. However, in our work, the number of taxa disclosed for four of the seven investigated phyla (Gastrotricha, Mollusca, Nemertea, and Xenacoelomorpha) was lower based on metabarcoding compared to morphological taxonomy. Surprisingly, for both gastrotrichs and nemerteans even the total estimated richness based on metabarcoding data was lower than the actual observed number of species obtained by morphological and molecular analyses of individuals. This discrepancy occurred for all 19 investigated sites supporting a consistent bias in assessing biodiversity using metabarcoding approaches for at least these phyla.

Several potential reasons exist that could explain the apparent discrepancy in species estimation using metabarcoding for certain phyla. One possible factor is the choice of the target gene(s) used for metabarcoding. Given the variability in the number of mutations in a gene within and among species, and variance in lineage histories, it is nearly impossible to establish a commonality among taxa. Even within the same marker (ribosomal or mitochondrial), extensive variation was observed within-species^19^, while in other instances, no variation was detected even among species^20^. With respect to this discrepancy, it is important to note that the analysis of individual specimens included both morphology and molecular analysis of 18S rRNA. In our analysis, metabarcoding employed highly conserved eukaryotic primers (V9 region), whereas individual specimens used less broadly conserved primers designed for invertebrates (V1–V2 regions). The cost of using highly conserved primers may represent a tradeoff between the loss of individual species for the amplification of phylogenetically diverse groups. Our observed low species richness for the metabarcoding relative to the analysis of individual specimens could simply reflect a lack of sequence divergence for the V9 region relative to the V1–V2 region in these phyla. However, comparisons of these two regions among complete published sequences for representatives of all phyla shows that the rate of divergence of V9 region is systematically higher than the V1–V2 region.

Regardless of the genetic region, additional possible factors could be the characteristics of these phyla that limit the availability of DNA templates. This could include relatively low body mass^21^, population density, extractability of both organisms and DNA, or ribosomal DNA copy numbers. The observation in nemerteans is unlikely to be due to low body mass, however, small population size is a possible factor. The opposite is true for gastrotrichs. Further, there is no evidence for a systematic bias against extractability for either nemerteans or gastrotrichs. With respect to the copy number of the ribosomal RNA gene arrays, while we do not have direct estimates of copy numbers for these taxa, they are known to be highly variable across species and unlikely to explain the observed phylum wide biases.

Only the richness of annelids and nematodes were significantly increased by metabarcoding (all associated p-values equal or lower than 0.0004). However, the richness of annelids is significantly higher only when the eOTU approach is applied (p-values equal or lower than 0.0001). The application of eOTU clustering analysis is intended to eliminate the effect of sequencing errors and variations among individual ribosomal repeats. By contrast, sequence variant methods are developed with specific pipelines to reduce such errors, they have demonstrated sensitivity and specificity as good as or better than eOTU methods^22,23^. The lack of difference in the phylogenetic diversity between eOTU and sequence variant datasets supports that eOTU clustering does not increase the actual species diversity, but only overestimates the number of biological units in the samples. For nematodes, richness was significantly different between metabarcoding and individual DNA taxonomy predictions obtained with the GMYC model (p-values equal or lower than 0.0004). However, the number of species estimated by that model was not statistically supported (*p* = 0.773). Moreover, the application of this species delineation model to the 18S rRNA locus has been shown to underestimate diversity in many meiofaunal taxa^24,25^.

Uneven differences in richness and incidence across taxa greatly affect the correlations with environmental parameters, especially when biodiversity is investigated at the community level. For example, biodiversity was significantly correlated to depth (*p* = 0.003) only when single specimens were collected by the taxonomists, likely due to a biased effort in investigating samples morphologically. Taxonomists are constrained to work on fresh sediments within a limited timeframe before samples are no longer suitable for morphological investigations. Therefore, the number of individuals investigated and species revealed by morphology from each depth may, not surprisingly, reflect the number of samples collected. This bias is avoided with the application of metabarcoding, which has the potential to provide an inclusive view of diversity regardless of the time invested on each sample. Preliminary surveys of diversity across sites using environmental DNA methods would be extremely helpful to drive, in advance, the focus of taxonomists toward specific sites with a higher and/or particularly interesting diversity. More evidence of bias by the taxonomic method applied is the strong correlation between biodiversity and the grain size of the sediment that was found only in the morphological dataset. Grain size is considered among the most important factors that affect meiofaunal biodiversity^8,26^, and such biases need to be removed in order to reach an inclusive understanding of the ecological effect in marine sediments.

Lastly, differences in the ecological correlations are particularly relevant when the same metabarcoding method (eOTU or sequence variant) is applied to either the focal or complete meiofaunal dataset. Meiofaunal organisms belong to diverse groups characterized by having intimate correlations with the surrounding environment^27^. Our results suggest the importance of taking into account the taxonomic group under investigation, without generalizing to higher taxonomic ranks or to the whole meiofaunal community. Currently, the majority of investigations in ecological and monitoring perspectives are performed on organisms that allow morphological study of preserved samples, such as nematodes, copepods, and kinorhynchs^28,29^. Neglecting soft-body organisms, however, will yield biased results that do not adequately characterize or represent the whole marine meiobenthic community.

In conclusion, the application of metabarcoding, compared to collecting single individuals, did not substantially improve estimates of biodiversity in our investigated samples, although metabarcoding is expected to disclose the taxonomy of specimens usually neglected by morphology-based taxonomists. Moreover, while morphological taxonomy allows identification at low taxonomic ranks (generally genus or species), most of the meiofaunal taxa represented in general reference libraries for DNA sequence data remain poorly classified and some represent misidentifications or contaminations^30^. Hence, although metabarcoding enables untangling of great phylogenetic diversity, it is not possible in most cases to classify species, and most remain known only at the phylum level^6,31^. Accurate low-level taxonomic placement of metabarcoding sequences improves many kinds of assessments of the structure and function of communities, and how these change over space, time, or environmental gradients. Nonetheless, the choice of the genetic loci, bioinformatics, and sampling protocol represent variables in metabarcoding^11,24,32^. As suggested by other works^33^ and the present study, employment of highly conserved primers likely yields uneven amplification efficiencies among PCR products. To overcome this, the integration of multiple taxon-specific primers is pivotal to ensure accurate estimates of biodiversity at the species level. Going forward, this issue may be addressed by a multi-barcode approach (i.e., using a combination of different genetic loci for each sample), which could help to improve taxonomic coverage and resolution of metabarcoding studies. To avoid primer biases and gain access to potential metabolic functions, metagenomics has the potential to provide a holistic view of the ecosystem diversity and function by disclosing genetic information from any genome present in the environment, including bacteria/archaea, eukaryotes, and viruses. However, a key step for the development of such an approach is building a reliable reference library of DNA sequences^6,34,35^. As such, the success of metagenomics (as well as metabarcoding) largely depends on the taxonomic diversity of sequences deposited in the reference library. Currently, there are active collaborations involving large groups of researchers to create better reference libraries across all groups^34,35^. This goal will only be achieved with the integrated effort of both morphology-based taxonomists and computational biologists^6,36^.

## Methods

### Sampling

Nineteen sites in Panama (collecting permit No SE/A-2-16) were sampled between February 22 and March 9, 2016 (Fig. 1; see additional tables for a list of the sampling sites^15^). From each sampled site, geographic coordinates were registered; salinity and depth were measured with, respectively, a VWR International Brand Hand Held Refractometer and a Suunto Zoop Dive computer, and sedimentological parameters were obtained. According to the Scientific Committee on Oceanic Research working group, salinity has to be unit-less, as the measurement is based on conductivity and is not precisely related to the mass of dissolved material^14^.

Sediment texture was evaluated by passing 1 g of sample (dried sediment) through a Microtrac Bluewave S54000 laser granulometer that uses light refraction technology. The determination of grain size by this equipment is based on the interaction between three red laser beams (*λ* = 780 nm) and the sediment particle. Sedimentological parameters were calculated with the software Microtrac FLEX 10.6.2, which uses Mie's compensation theory to describe the effect of spherical particle shapes on light. As recommended by Tanner et al.^37^, final sedimentological values were calculated via the Method of Moments, a parameter estimation technique that involves equating sample moments with theoretical moments. Textural classification of the sediment was based on size ranges and names were assigned according to the Wentworth scale^38^.

Samples consisted of at least two replicates, each of which consisted of about 2–4 L of sediment from the upper 5 cm of the sea bottom collected over a homogeneously sandy area (about 2–4 m^2^), sampled by scooping the top layer of sand with a jar or a bucket. The sediments were collected by hand from littoral beaches (depth: 0–0.5 m; 9 sites), by apnea freediving in the sublittoral zone (depth: 1–4 m; 6 sites); and with SCUBA divers offshore (depth: 6–16 m; 4 sites). Immediately after collection, samples were taken to the Achotines Bay Laboratory Facility (www.iattc.org/AchotinesLab/AchotinesResearchFacilitiesENG.htm), where they were processed immediately. Each sample was divided into two parts; one of which was used for investigations of single individuals, and the other for metabarcoding analyses.

### Meiofauna extraction

All meiofauna specimens used for morphological assessment were extracted from sediment collected from the 19 sites using an isotonic MgCl_2_ solution decantated by hand through 45 μm or 63 μm sieves. Total meiofauna for metabarcoding were extracted using the same MgCl_2_ decantation techniques^39^ through a mesh size of 42 µm and immediately preserved in ethanol 90%.

### Investigation of morphotypes

Live material was studied using stereo and compound light microscopes Microscopes used were: optical Leitz Orthoplan 2 microscope with Canon Rebel T2i digital camera (Fig. 2a, c, e, f); differential interference contrast Leitz dialux 20 microscope with DS-Fi1 Nikon digital camera driven by the Nikon NIS-F v.4.0 software (Fig. 2b); optical Nikon Eclipse microscope with Cool Pix 4300 Nikon digital camera; optical Zeiss Axiostar microscope with Power Shot G6 Canon digital camera (Fig. 2d, g); differential interference contrast Nikon Eclipse 80i microscope with Canon EOS 5D mk III digital camera (Fig. 2h). Individuals from each identified morphotype (maximum 20 individuals per morphotype) were preserved in ethanol 90% for further molecular analyses. The taxonomic effort was focused on species belonging to the phyla Annelida, Gastrotricha, Mollusca, Nematoda, Nemertea, Platyhelminthes, and Xenacoelomorpha (Fig. 2). We followed the recent phylogenetic hypothesis and consensus that sipunculans are annelids, and include results from both taxa together in the phylum Annelida^40^.

### DNA taxonomy

*DNA extraction, amplification, and sequencing*: DNA was extracted from single individuals at the Laboratories of Analytical Biology of the Smithsonian Institution National Museum of Natural History (export permits No SEX/A-32-16; April 27th, 2016) using an automated DNA extraction system (AutoGen, Inc). Tissue digestion was performed at 56 °C in a shaker incubator with 150 µL of M2 buffer mixed to 150 µL of M1 buffer and Proteinase K. DNA extraction was performed using the Autogen Prep 956 Extractor. The DNA was eluted in 100 µL of R9 buffer^25^ and is currently stored at the biorepository of the Smithsonian National Museum of Natural History (Washington D.C.) in tubes marked with unique barcodes. Manufacturer buffers were provided by Autogen, Inc.

Polymerase chain reaction (PCR) was performed on the DNA of single individuals. Specifically, amplification of the V1–V2 region of *18S rRNA* was carried out using the 18S EukF forward primer [AACCTGGTTGATCCTGCCAGT] and the SR7 reverse primer [GTTCAACTACGAGCTTTTTAA]^39^. Either a 25 µL final volume with 50 mM Tris–HCl pH 9.1, 16 mM (NH_4_)2SO_4_, 3.5 mM MgCl_2_, 150 mg/mL bovine serum albumin (BSA), 0.5 mM of each primer, 160 mM of each dNTP, and 0.05 U/μL of KlenTaq polymerase (AB Peptides, Inc.); or a 10 μL final volume with 3 pmol of each primer, 500 μM dNTPS, 3 mM MgCl, 0.25 mg/μL BSA, and 0.05 U/μL of Biolase^TM^ DNA polymerase (Bioline, Inc.) with manufacturer provided buffers was the input to the thermal cycler. Thermal cycler parameters for the Biolase^TM^ DNA polymerase included an initial denaturing step of 95 °C for 3 min, followed by 40 cycles of 95 °C for 30 s, 50 °C for 60 s, 72 °C for 60 s, and a final extension step of 72 °C for 5 min. Thermo cycling using the KlenTaq polymerase comprised an initial 3-min denaturation at 95 °C, followed by 35 cycles of 30 s at 95 °C, 30 s at 52 °C, 45 s at 72 °C. The cycling ended with a 7-min sequence extension at 72 °C. PCR products were purified with Illustra Sephadex columns and then used for cycle sequencing with dye-terminators using BigDye chemistry (Perkin-Elmer) and standard cycling (4 min denaturation at 96 °C, followed by 25 cycles of 10 s at 96 °C, 5 s at 50 °C, and 4 min at 60 °C). Cycle-sequenced products were run on an ABI 3730xl 96-well capillary sequencer at the Laboratories of Analytical Biology at the Smithsonian National Museum of Natural History.

### Genetic alignment and phylogenetic inference

Sequences obtained from single organisms, about 600 bp long (*18S rRNA*, region V1 and V2), were checked for possible contaminations or misidentifications using BLAST. Sequences organized by phylum were aligned using MAFFT v7.017^41^, by implementing the Q-INS-I algorithm known as the optimal strategy for ribosomal markers^42^. Unique sequences were used to obtain ultrametric trees. The selected model of evolution for the phylogenetic reconstructions of each taxon was general time-reversible-plus-gamma-distribution (GTR+G) chosen by hierarchical likelihood ratio tests in ModelGenerator v. 2.145^43^. The model of evolution was run to reconstruct phylogenetic trees with BEAST 2.2.1^44^ to obtain Bayesian ultrametric trees. Beast was run for 100 million generations and a tree was sampled every 10,000 generations. The summarized trees were calculated with the program TreeAnnotator (part of the BEAST package) after discarding the first 20% of the trees as burn-in. As outgroups for rooting, we used sequences obtained in the present work from the phylogenetically closest clade.

### Test for evolutionary independent entities and OTUs

In order to quantify evolutionary independent entities, the ultrametric trees obtained with BEAST were used as input for the single-threshold GMYC approach in R 2.15.3^45^ with the package *splits* (http://splits.r-forge.r-project.org/)^46^. The GMYC method is a likelihood method for delimiting species under coalescent theory by fitting within-species and between-species branching models to reconstructed ultrametric gene trees^4^.

To obtain OTUs, the amplified *18S rRNA* sequences were concatenated into a single FASTA file to mimic the input of a processed metabarcoding dataset. OTUs are based on sequence similarity^47^. The sequences were clustered into OTUs using the QIIME open-reference OTU picking pipeline and the SILVA (release 128) 99% identity SSU ribosomal sequence reference database^47^.

### Metabarcoding

*DNA extraction, amplification, and sequencing*: DNA was extracted for metabarcoding from the sediments samples, for each of the 19 locations, at the Laboratories of Analytical Biology, Smithsonian Institution National Museum of Natural History. Each sample was vortexed for 10 s after which 10 mL were transferred to 2-mL tubes. The ethanol was evaporated in a 60 °C vacuum spinning centrifuge. 0.5 mL of extraction buffer (0.1M Tris–HCl, 0.1M NaCl, 0.1M EDTA, 1% SDS and 250 μg/mL proteinase K)^48^ was added to the dry sample which was then incubated for 2–12 h at 56 °C. The samples were then re-suspended by vigorous pipetting and all the liquid was transferred to the microbead-tube of the commercial PowerSoil® DNA Isolation Kit (Mobio Carlsbard, CA), according to the manufacturer’s protocol. DNA was eluted in 100 µL water and quantified prior amplification.

DNA amplification, purification, and sequencing for metabarcoding analyses were all conducted following the Earth Microbiome Project protocol for *18S rRNA* (region V9) Illumina Amplicon^47^. Specifically, the *18S rRNA* (region V9) was amplified using Illumina_Euk_1391f forward primer [GTACACACCGCCCGTC] and Illumina_EukBr reverse primer, barcoded [TGATCCTTCTGCAGGTTCACCTAC]. The input to the thermal cycler was a final volume of 25 µL including 12 µL of PCR-grade water, 10 µL of PCR master mix, 1 µL of forward primer (5 µM), 1 µL of reverse primer (5 µM), and 1 µL of template DNA. Thermal cycler conditions included an initial denaturing step of 94 °C for 3 min, followed by 35 cycles of 94 °C for 45 s, 57 °C for 60 s, 72 °C for 60 s, and a final extension step of 72 °C for 10 min. The PCR products were purified with Rapid PCR Purification System Kit Marligen Bioscience, Inc.

### Construction of eOTU and sequence variant tables

To reconstruct eOTU tables, forward and reverse reads were merged using PEAR V0.9.6; bases with a *phred* quality score less than Q3 were trimmed^16^. The merged sequencing reads were clustered into operational taxonomic units using the QIIME open-reference OTU picking pipeline and the SILVA (release 128) 99% identity SSU ribosomal sequence reference database^47^. Within the pipeline, reverse-strand matches were allowed and a percent identity threshold of 99 was used for UCLUST clustering^49^. The final result of the QIIME pipeline is an OTU table with per sample counts of each eOTU^16^. Sequence variant tables were constructed following the DADA2 V1.4 pipeline^22^. Forward and reverse reads were truncated to 130 base-pairs on quality profiles. Reads were then de-replicated and binned into unique sequence variants. Final sequence variants across the samples were then inferred using a parametric error model customized for each sample dataset. Forward and reverse reads were then merged and a ‘sequence table’ was constructed with per sample total counts for each sequence variant (analogous to the classical OTU table). Chimeric sequence variants were removed from the final table^16^.

### Taxonomic classifications of eOTUs and sequence variants

Each sequence from the dataset was compared to the SILVA 18S reference set using BLAST. The taxonomy from the top five BLAST hits were then compared and the best consensus taxonomy was obtained. BLAST hits were only considered if the percent identity of the match falls within 0.5% identity of the top hit and if the alignment of the hit spans >120 bp. Taxonomic identifications were made to the species, family, and phylum level if the percent identity of the best hit was >97, 93, and 90, respectively. These threshold values were arbitrarily chosen according to diverse literature sources and investigations that were mostly based on mock communities of selected taxa^50,51^. To validate the taxonomic classifications of the sequences, a phylogenetic tree was constructed for each dataset and the phylum-level taxonomic identities for each clade were examined. Previously unidentified sequences were dubbed a certain phylum when belonging to a supported clade including at least five other sequences, all them belonging to that phylum.

A constrained phylogenetic tree was reconstructed to show the taxonomic composition of the whole meiofaunal dataset in the sampled area. First, sequence variants were grouped in their respective phyla based on the taxonomic classifications. Separated alignments were performed with Uclast and PyNAST method, a python implementation of the NAST alignment^47^. Maximum Likelihood phylogenies were reconstructed using RAxML v8.2^52^. Finally, the separate phyla trees were combined into a single tree. The topology of the final tree was constrained following the animal phylogeny proposed by Dunn et al.^53^

### V1–V2 and V9 primer region comparison

Primer locations were identified from each of the metazoan *18S rRNA* sequences in the SILVA SSU database using blastn. Variable regions (V1–V2 and V9) were then extracted and separated into multi-fastas for each phylum. Each respective FASTA was then aligned using clustal omega with an option to output a full distance matrix. Average percent identity was then calculated for each phylum–primer pair using custom python scripts^16^.

### Statistical analyses

Our goal was testing whether different taxonomic methods provide significant differences in revealing the diversity of the seven focal phyla, and how possible differences affected the correlation with environmental parameters. The significance of explanatory variables in all regression analyses was based on two-sided tests of the associated regression coefficients. The effect of the methodology used was evaluated through three different types of community descriptors, which were included as response variables in the different sets of models.

#### Richness

This was measured as the number of unique units (= putative species) from each of the five methods applied on (a) single individuals, namely morphotypes obtained with morphological taxonomy, evolutionary independent entities obtained with GMYC model, and OTUs; and on (b) environmental samples, namely eOTU and sequence variant. Analyses of data obtained with metabarcoding were performed for the seven focal phyla as well as the total number of eOTUs or sequence variants for all the meiofauna groups present in the sediments.

#### Species composition

Differences in community structure (*β*-diversity) between samples were measured for units obtained by every single method using the *Jaccard* dissimilarity index^54,55^ calculated with the function *beta.pair* included in the package *betapart* 1.3 of R statistical software system^56^. Biological units obtained by each of the five methods were organized in presence/absence (incidence) datasets.

#### Phylogenetic diversity

A quantitative measure of phylogenetic diversity, defined as the minimum total length of all the phylogenetic branches required to span a given set of taxa on the phylogenetic tree, was measured^57^. Diversity and sorting at the phylogenetic level were measured on ultrametric BEAST trees calculated for the focal groups. Phylogenetic diversity was calculated with the function *pd* whereas phylogenetic sorting was estimated using the standardized effect size of the MPD, equivalent to 1 − Nearest Relative Index (NRI) and MNTD^58^, calculated with the functions *ses.mpd* and *sesmntd*, included in the R package *picante* 1.6-2^59^. Datasets used are unique genetic sequences obtained from single individuals and environmental samples.

### Statistical models

*Richness estimation*: In order to estimate the species richness for our sampled locality, we computed the species estimator functions *chao2* and *jack1* in the R package *fossil*^55,60,61^. These functions apply the algorithms *Chao* and *Jackknife* using incidence data. In fact, the number of individuals in our qualitative samples did not reflect the natural population abundance. Moreover, measuring abundance based on the number of genomic reads may be misleading because body size and cell numbers differ between species and individuals, the number of rRNA gene copies can vary within-species^62^, the occurrence of extensive intragenomic variation^63^, and possible PCR biases. The seven focal phyla and complete meiofaunal community were considered for the whole investigated area and sorted by three depth descriptors (littoral: 0–0.5 m; sublittoral: 1–4 m; and offshore: 5–16 m). To evaluate richness estimation, we did not consider datasets including evolutionary independent entities, OTUs, and eOTUs for the following reasons: the number of entities was not supported statistically for most phyla; the richness obtained via OTU analysis was comparable to the evaluation of distinct morphotypes, thus, we considered morphotypes in order to include also additional units, from which DNA amplification and/or sequencing failed. Lastly, in order to be more conservative in species estimation, we chose sequence variant approach which estimates a lower richness than eOTU.

### Effect of the method on richness

To identify significant predictors of genetic richness (response), a generalized linear mixed-effects model was pursued. More specifically, a Poisson (log-linear) model form was assumed, a model form that is theoretically appropriate for count data such as species richness. Using the Poisson model form, our full model included a single random effect for the 19 station locations, and several fixed effects including a continuous variable for sample size; continuous ecological variables such as depth, salinity, grain size, sorting, skewness, and kurtosis; and a categorical variable for taxonomic method. The Poisson model form has a fairly restrictive assumption that the response follows a Poisson distribution (equal mean and variance), and our particular data did show evidence of overdispersion. Thus, the Negative Binomial model form, an alternative to the Poisson model, was pursued as this model, considered a variant of the Poisson form, included an additional parameter to account for such overdispersion (overdispersion parameter = 1.7). Furthermore, based on a likelihood ratio test, the negative binomial model form (for our best model) provided a statistically significant improvement over a comparable Poisson model (Likelihood ratio *χ*^2^ = 441.86, df = 1, *p* < 0.0001).

All model fitting was performed using the package *glmmADMB*^64^ in the R statistical software system. Initial models (assuming a Negative Binomial form) considered all main effects and interactions between continuous variables (size, ecological variables) and categorical variable distinguishing species identification method. Because, based on our fitted model, we identified a statistically significant interaction effect (Wald *χ*^2^ = 153.01, *p* < 0.0001), we pursued a post-hoc analysis to compare levels of combinations of our categorical variables (phylum and methodology), or factors. To do this, we used the package *lsmeans*^65^ to calculate predicted values of each combination of factors. So, for our two factors, phylum and methodology, with 7 and 5 levels, respectively, we have 35 different combinations of factors. Then, for these 35 combinations, using a Tukey procedure for multiple comparisons to ensure a fixed family-wise error rate (*α* = 0.05), we performed all pairwise comparisons of predicted values^66^.

### Effect of the sample size and ecological parameters on richness

Because the values related to the sample size from single individuals (number of specimens or genetic sequences) and environmental samples (number of genetic reads) were not comparable, we evaluated the effect of the sample size and ecological variables on richness by performing separate analyses. To identify significant predictors of richness (response), a generalized linear mixed effect model was pursued. More specifically, a Poisson (log-linear) model form was assumed as it is theoretically appropriate for count data such as species richness^66^. In this case, no evidence of overdispersion (an assumption of the Poisson model form) was observed and subsequent fitting of a negative binomial model form to the data yielded a dispersion parameter estimate >400, an indication that the original Poisson assumption holds (and the negative binomial model form is not needed).

For the metabarcoding data associated with the whole meiofaunal community (not restricted to focused phyla), we followed the same basic approach as described above. However, for these data, we observed evidence of overdispersion and a negative binomial model form was assumed^66^.

### Analysis of community structure

For models on community composition using matrices of *Jaccard* pairwise differences as a response variable, we assessed the percentage of the variability in community composition observed across samples using a permutational multivariate analysis of variance applied on distance matrices (function *adonis2* in R package *vegan* 2.2-1;^67^). In these models, we included continuous environmental variables to test the significance of differences between sediments in structuring differences in community structures. Moreover, meiofaunal community dissimilarities were calculated within and among different depths as described above using the R package *betapart*, and the functions *beta.pair* and *beta.multi*^56^.

### Analysis of phylogenetic diversity

To analyze the relationship between phylogenetic diversity and both single animal / metabarcoding methodology and phylum, we use a mixed-effects linear model and analysis of variance (ANOVA). For our model, phylogenetic diversity was designated as our continuous response, and our explanatory variables included categorical fixed effects for sample methodology and phylum, the interaction between methodology and phylum, ecological variables, and a random effect for station location. All model fitting was performed package *nlme* in the R statistical software system^45^. We note that the model, as specified above, did yield significantly non-Normal model residuals. To remedy this, we transformed the response and reran the model using the square-root of the phylogenetic diversity. This model specification met all ANOVA/regression assumptions (errors Normally distributed with constant variance), and a Levene test showed no evidence of non-constant variance between groups (*F* = 1.23, *p* = 0.225). For the analysis regarding the MPD, the transformation of the phylogenetic diversity did not remedy the violation of the regression assumption. So, in light of the heavy-tailed (non-Normal) residuals, we implemented a robust regression model form, which is less restrictive in terms of assumptions. Such a model was fit using the package *robustlmm* in R statistical software^68^.

### Data availability

All data supporting the findings of this study are available within the article and its supplementary information files, which can be found at 10.6084/m9.figshare.6790640^15^. Sequence data that support the findings of this study have been deposited in GenBank with the accession codes MH302536–MH303208 for sequences obtained from single individuals, sequences obtained via metabarcoding are in the SRA database with accession code SRP142495. Additional outcomes that support results obtained by comparing the 18S rRNA V1–V2 region with 18S rRNA V9 region are available in GitHub with the identifier 10.5281/zenodo.1308829^16^. In consideration of partial funding by GOMRI, data are publicly available through the Gulf of Mexico Research Initiative Information & Data Cooperative (GRIIDC) at https://data.gulfresearchinitiative.org (10.7266/n7-szj8-1531).

### Code availability

Computer codes used for bioinformatics analyses that support the findings of this study are available in GitHub at 10.5281/zenodo.1308829^16^. Computer codes used for statistical analyses that support the findings of this study are available from the figshare record associated with this publication at 10.6084/m9.figshare.6295061^66^.
