# Author Correction: Biodiversity estimates and ecological interpretations of meiofaunal communities are biased by the taxonomic approach

**DOI:** 10.1038/s42003-018-0249-6

**Published:** 2018-12-20

**Authors:** Francesca Leasi, Joseph L. Sevigny, Eric M. Laflamme, Tom Artois, Marco Curini-Galletti, Alberto de Jesus Navarrete, Maikon Di Domenico, Freya Goetz, Jeffrey A. Hall, Rick Hochberg, Katharina M. Jörger, Ulf Jondelius, M. Antonio Todaro, Herman H. Wirshing, Jon L. Norenburg, W. Kelley Thomas

**Affiliations:** 1 Department of Biology, Geology and Environmental Science, University of Tennessee at Chattanooga, 615 McCallie Avenue, 37403 Chattanooga, TN USA; 2 Hubbard Center for Genome Studies, Department of Molecular, Cellular, and Biomedical Sciences, University of New Hampshire, 35 Colovos Road, 03824 Durham, NH USA; 3 Department of Mathematics, Plymouth State University, MSC29, 17 High Street, 03264 Plymouth, NH USA; 4 Centre for Environmental Sciences, Hasselt University, Campus Diepenbeek, Agoralaan Gebouw D, 3590 Diepenbeek, Belgium; 5 Dipartimento di Medicina Veterinaria, University of Sassari, via Muroni 25, 07100 Sassari, Italy; 6 Departmento de Sistemática y Ecología Acuática, El Colegio de la Frontera Sur, Unidad Chetumal, Av. Centenario Km. 5.5 Chetumal Quintana Roo, 77014 Chetumal, Mexico; 7 Centro de Estudos do Mar, Universidade Federal do Paraná, Av. Beira-Mar, s/n, Pontal do Sul, PO Box 61, 83255-976 Pontal do Paraná, PR Brazil; 8 Department of Invertebrate Zoology, Smithsonian National Museum of Natural History, 10th St. & Constitution Ave NW, 20560 Washington, DC, USA; 9 Department of Biological Science, University of Massachusetts Lowell, Olsen Hall 414, 198 Riverside St., 01854 Lowell, MA USA; 10 Department of Biology, Ludwig-Maximilians–University of Munich, Großhaderner Str. 2, 82152 Planegg-Martinsried, Munich Germany; 11 Swedish Museum of Natural History, POB 5007, SE-104 05 Stockholm, Sweden; 12 Department of Life Sciences, University of Modena & Reggio Emilia, Via G. Campi 213/d, 41125 Modena, Italy

**Keywords:** Biodiversity, Zoology, Genomics, Classification and taxonomy

Correction to: *Communications Biology*; 10.1038/s42003-018-0119-2; published online: 14 August 2018

In the original published version of the article, the acknowledgements incorrectly omitted a statement acknowledging the availability of public data through the authors’ funding from the Gulf of Mexico Research Initiative. This information was also missing from the Data Availability statement. In addition, the original version of the acknowledgements did not accurately reflect the relative funding to author Francesca Leasi from multiple sources. These errors have been corrected in the HTML and PDF versions of the article.

